# Notes and News

**Published:** 1893-06-03

**Authors:** 


### Notes and News.

Collected and Communicated.

Amongst the numerous offerings to H.R.H. the Duke of York and the Princess May is to be a costly gift from the citizens and inhabitants of London. The Lord Mayor heads the committee list of which Lord Rothschild, Lord Brassey, and Alderman Sir David Evans are amongst the many influential members. All inhabitants of London can join in this offering, which will be doubtless, a much valued memento, as coming from the people amongst whom the Duke of York and the Princess May have spent a large portion of their lives.

We regret to announce the death of Dr. W. B. Hadden, who closed a most promising career on May 26th at the early age of thirty-seven. His loss will be much felt by St. Thomas's Hospital, the Children's Hospital, Great Ormond Street, and the Nurses' Co-operation Society. He qualified as a member of the Royal College of Surgeons in 1877, took his M.B. (London) degree in 1878, that of M.D. (London) in 1879, and became a Fellow of the College of Physicians in 1881. Dr. Hadden's career is further referred to in another portion of the paper. Dr. Hadden was a courteous and loyal friend, an able and accomplished physician, and a man whose loss will be felt and mourned by everyone who had the privilege of being a fellow worker with him.

Lord Rothschild has consented to preside at the festival dinner of the Royal Hospital for Diseases of the Chest, City Road at the Hotel Metropole, on Tuesday, the 20th of June. We regret to state that the expenditure for the past year exceeded the income by £1,024, and that the present obligations amount to £1,632. Therefore, unless a liberal response is made to the present appeal, the council may be compelled seriously to consider the necessity of still further reducing the number of in-patients which the hospital is capable of receiving within its walls. This would be a grave misfortune to the suffering poor of the metropolis generally, and more especially to those of them who reside in the immediate neighbourhood of the hospital, and we venture to hope that generous help will be extended to this the oldest institution of its kind in Europe.

At the Quarterly General Court of Governors of the Middlesex Hospital, held on 25th ult., the expenditure of £2 098 on new teak floors, painting, and repairs was authorised, and authority was also given for the sale of $15,000 United States Bonds in order to repay an advance of £4,000 from the bankers for current expenses. It was announced that the Weekly Board had decided to close the hospital entirely from 1st July to 18th September, to enable the contractors to carry out the extraordinary repairs and other works.

The Royal National Hospital for Consumption has just issued an interesting little collection of reproductions of photographic views of the hospital. This will provide those interested with a far better idea of the arrangements of the institution than any written description unillustrated can do. The Ventnor Consumption Hospital has been so planned as to give the best possible opportunities for the favourable treatment of the terrible disease so especially prevalent in this country. The hospital, as illustration No. 1 shows, is practically a row of separate well-appointed dwelling-houses. It is built close to the sea, with charming views, and the site in fact is all that could be wished. We advise our readers to send to the secretary for a copy of the very interesting illustrated pamphlet, which cannot fail to rouse readers to the claims and requirements of this necessarily costly national institution.

The hospital at Luxor, Egypt, was established by Mr. John Mason Cook, of the well-known tourist firm, who supplied the largest part of the funds required for buying the ground on which the building stands and erecting it. Mr. Brunner, M.P., and others who had visited Luxor, contributed what was wanting. The hospital is situated behind the Luxor Hotel, the structure forming a hollow square, with a garden in the interior. Roses flourish in the garden, and shoots of eucalyptus trees are the beginnings of what will become a pleasure to the eye and a preventive of fever. The hospital was opened by the late Khedive in February, 1891, when Mr. J. M. Cook made a free gift of it to the inhabitants of the district, promising to contribute to its support, and hoping to induce charitable persons to aid them. The Khedive declared his gratification at being asked to dedicate the Luxor Hospital to the poorer inhabitants of Upper Egypt.

There is in New York a very useful little society known as the Ladies' Health Protection Society. At a meeting of the New York Academy of Medicine a resolution was handed in by this society to the effect that "the cremation of street garbage and city refuse is the only safe and practicable method of disposing of the same, and the Association requests the city authorities to at once consider the subject." The destruction of city refuse by fire naturally commends itself to the common-sense persons, but the impracticable method of carrying this into effect as suggested by all London local authorities is not likely to be forgotten. Indignation at a decree which required all rubbish to be burnt within the crowded tenements of the poor was very natural, and, before a visitation of cholera comes upon us, it is to be hoped that the authorities will have thought out some feasible cremating grounds for the city rubbish.

